# Functionally Antagonistic Transcription Factors IRF1 and IRF2 Regulate the Transcription of the Dopamine Receptor D2 Gene Associated with Aggressive Behavior of Weaned Pigs

**DOI:** 10.3390/biology11010135

**Published:** 2022-01-14

**Authors:** Jing Zhao, Siyuan Gao, Yanli Guo, Qinglei Xu, Mingzheng Liu, Chunlei Zhang, Meng Cheng, Xianle Zhao, Allan P. Schinckel, Bo Zhou

**Affiliations:** 1 College of Animal Science and Technology, Nanjing Agricultural University, Nanjing 210095, China; 2019105039@stu.njau.edu.cn (J.Z.); 2018105082@njau.edu.cn (S.G.); 2019105040@njau.edu.cn (Y.G.); 2019205004@njau.edu.cn (Q.X.); liumingzheng@stu.njau.edu.cn (M.L.); 2020105039@stu.njau.edu.cn (C.Z.); 2020805124@stu.njau.edu.cn (M.C.); 2020805123@stu.njau.edu.cn (X.Z.); 2 Department of Animal Sciences, Purdue University, West Lafayette, IN 47907-2054, USA; aschinck@purdue.edu

**Keywords:** porcine, aggressive behavior, DRD2, IRF1, IRF2, SNP, ChIP

## Abstract

**Simple Summary:**

Aggressive behavior in pigs after mixing is an issue of animal welfare. This trial was conducted to evaluate the impact of the dopamine receptor (DR) D2 gene on aggressive behavior in pigs. We found that the core promoter region of the DRD2 gene is located in −2212~−1127 bp. A single-nucleotide polymorphism, rs1110730503, for the DRD2 gene is associated with aggressive behavior in pigs and influences the binding of the transcriptional factor interferon regulatory factor (IRF) 2. The transcriptional factor IRF1 upregulates the expression of the DRD2 gene; however, IRF2 downregulates the gene’s expression. IRF1 and IRF2 are functionally antagonistic to each other. Aggressive behavior in pigs is impacted by the DRD2 gene and its expression. These results provide insight into the genetics and neurophysiology of aggressive behavior in pigs.

**Abstract:**

Aggressive behavior has negative effects on animal welfare and growth performance in pigs. The dopamine receptor D2 (DRD2) has a critical neuromodulator role in the dopamine signal pathway within the brain to control behavior. A functional single-nucleotide polymorphism (SNP), rs1110730503, in the promoter region of the porcine DRD2 gene was identified, which affects aggressive behavior in pigs. A chromatin immunoprecipitation (ChIP) assay was used to identify the interactions between interferon regulatory factor 1 (IRF1) and IRF2 with the DRD2 gene. The overexpression or knockdown of these two transcription factors in porcine kidney-15 (PK15) and porcine neuronal cells (PNCs) indicate that the binding of IRF1 to DRD2 promotes the transcription of the DRD2 gene, but the binding of IRF2 to the DRD2 gene inhibits its transcription. Furthermore, IRF1 and IRF2 are functionally antagonistic to each other. The downregulation of DRD2 or upregulation of IRF2 increased the apoptosis rate of porcine neuroglial cells. Taken together, we found that transcriptional factors IRF1 and IRF2 have vital roles in regulating the transcription of the DRD2 gene, and rs1110730503 (−915A/T) is a functional SNP that influences IRF2 binding to the promoter of the DRD2 gene. These findings will provide further insight towards controlling aggressive behavior in pigs.

## 1. Introduction

To improve the efficiency of space utilization and management, regrouping is a common practice in intensive pig farms [1]. However, because pigs are social animals, unfamiliar pigs often fight with each other after mixing to obtain feed, space, and other resources until a new social hierarchy has been reestablished [1]. A previous study showed that growth traits were related to behavioral traits in pigs [2]. Aggressive pigs have a greater probability of being kept for breeding than docile pigs in a pen, because they consume more feed and grow faster [3]. Therefore, in recent decades commercial pig breeds have increasingly become more aggressive with selection for increased growth performance [3]. In addition, aggressive behavior causes serious physical injury to pigs, increases the risk of disease, and reduces the level of animal welfare [4]. Since aggressive behavior traits are difficult to assess for breeding selection in commercial pig farms, one alternative means of reducing aggressive behavior is to identify and select pigs based on molecular genetic markers of aggressive behavior [5,6].

Single-nucleotide polymorphisms (SNPs) located in noncoding RNA genes are classified functional SNPs which affect different biological processes and continually confer risk for multifactorial diseases [7]. Emerging evidence indicates that SNPs regulate gene expression levels by modulating the binding of transcription factors, transcriptional enhancers [8] or microRNAs, or directly changing the protein structure because of missense mutation [9,10]. For example, SNP rs4680 in the catechol-O-methyltransferase (COMT) gene is related to schizophrenia in humans, because the G > A mutation of rs4680 decreases the expression level of the COMT gene [11].

Dopamine (DA) is an important neurotransmitter in the central nervous system, and the majority of DA synthesis occurs directly from tyrosine [12]. It regulates movement, motivation and reward, learning and memory, emotion, addiction, and endocrine [13]. The DA receptor family, including dopamine receptors (DR) D2, DRD3, and DRD4 [14,15], are the most studied DA receptors. DRD2 influences the signal transmission pathway through two different ways: (1) DRD2 binds to the G protein to stimulate DRD2 activity [16], which inhibits adenylate cyclase and decreases cyclic adenosine monophosphate (cAMP) levels [16,17], and downregulates protein kinase A (PKA) [18]; (2) DRD2 induces the signaling complex of threonine kinase 1 (AKT1), protein phosphatase 2A (PP2A) and β-arrestin2 to activate glycogen synthase kinase-3β (GSK-3β) [19], transduce dopamine-dependent behavior, and downregulate PKA activity [20]. A study on the Caucasian population found that individuals with homozygous TT of C957T polymorphism in DRD2 gene, or a variable number of tandem-repeat (VNTR) allele, 7 repeats in the DRD4 gene, had abnormal impulsivity [21].

A series of proteins that specifically bind to DNA called transcription factors regulate the process of transcription from DNA to mRNA [22]. SNPs located in the transcription factor’s binding region of a gene may affect the binding and the gene expression level [23]. The transcription factors interferon regulatory factor 1 (IRF1) and interferon regulatory factor 2 (IRF2) belong to the nine-member interferon regulatory factor (IRF) family [24]. Previous studies have shown that IRF1 acts as an activator, while IRF2 acts as a repressor [25,26]. Both IRF1 and IRF2 also regulate the production and function of immune cytokines [27]. A previous study found that the mesolimbic dopamine system (MLDS) directly increased the levels of inflammatory cytokines, which ultimately caused the development of mental illness and other medical diseases [28]. The increase of cytokines, such as interleukin (IL)-6, tumor necrosis factor (TNF)-α, and IL-1β, induce aggressive behavior in humans [29].

Our bioinformatics prediction analysis found that transcription factors IRF1 or IRF2 could bind to the promoter of the porcine DRD2 gene. Therefore, we hypothesized that the SNPs located in the promoter region of porcine DRD2 gene changed the binding sites of transcription factors IRF1 or IRF2, which could influence the aggressive behavior of pigs through the DA signaling pathway and immune pathway. The objective of this study is to identify the SNPs of the DRD2 gene that are involved in the regulation of IRF1 and IRF2 binding, as well as to explore the molecular regulation mechanism of the DRD2 gene.

## 2. Materials and Methods

This study was approved by the Animal Care and Use Committee of Nanjing Agricultural University (SYXK Su 2017-0027).

### 2.1. Animals and Aggressive Behavior Analysis

The animal trial was carried out at Huaiyin pig breeding farm, Huai’an, Jiangsu Province. A total of 500 piglets were randomly selected from 65 litters of multiple sows. Piglets were weaned at 35 days of age, then nine or ten weaned piglets from different litters were mixed in pens of dimension 2.5 m × 2.2 m. The pens were equipped with slatted floors, stainless steel feeders, and nipple drinkers to allow ad libitum access to feed and water. The pigs’ behavior was recorded for 72 h after mixing, with a digital video recording system (Hikvision Digital Technology Co. Ltd., Hang-zhou, China). All pigs were marked with different numbers on their backs using a spray paint, for individual identification in the video record. Aggressive behavior was assessed by observing the video and recording the frequency and duration of active attacks, bullying, and stand-off behaviors for each pig in a pen for 72 h after mixing [30]. A composite aggressive score (CAS) was adapted as follows: CAS = frequency of active attack + 0.07 × duration of active attack[s] [30]. The two most aggressive pigs and the two least aggressive pigs were selected in each pen according to their CAS value for further association analysis.

### 2.2. Potential SNP Identified

The total DNA was extracted from ear tissue of piglets by standard phenol/chloroform method (Roche, Beijing, China). Specific primers (Supplementary Table S1) were designed with Primer 5.0 software to amplify porcine DRD2 gene 5′-UTR region. PCR reactions were performed using rTaq and LATaq Master Mix (Takara, Dalian, China). The amplified PCR products were sequenced. The software DNAMAN 8.0 and Chromas 2.6.4 were used to analyze the difference between the most aggressive and the least aggressive pigs. Finally, aggressive behavior indicators of pigs were analyzed using the GLIMMIX procedure in SAS.

### 2.3. Bioinformatics Analysis

The gene sequences for DRD2 (ENSSSCG00000015048), IRF1 (ENSSSCG00000014277), and IRF2 (ENSSSCG00000015782) were downloaded from the NCBI (https://www.ncbi.nlm.nih.gov/) (9 July 2020) and ENSEMBL (http://asia.ensembl.org/index.html) (9 July 2020) websites. The upstream sequence of the porcine DRD2 gene was used for promoter prediction by online Neural Network Promoter Prediction (http://www.fruitfly.org/seq_tools/promoter.html) (9 July 2020) [31] and Promoter 2.0 server (http://www.cbs.dtu.dk/services/Promoter/) (9 July 2020) [32]. Transcription factor binding sites were predicted using JASPAR (http://jaspar.genereg.net/) (9 July 2020) and ALGGEN-PROMO (http://alggen.lsi.upc.es/cgibin/promo_v3/promo/promoinit.cgi?dirDB=TF_8.3) (9 July 2020) [33,34]. The CpG islands in the DRD2 promoter were predicted by the CpG Island Searcher (http://www.protocol-online.org/cgi-bin/prot/view_cache.cgi?ID=2943) (9 July 2020) and Meth Primer (http://www.urogene.org/methprimer/) (9 July 2020) [35].

### 2.4. Plasmid Construction

Deletion fragments (P1: −2212/+66; P2: −1127/+66; P3: −527/+66) and wild-type (containing the transcription factor binding sites) DNA fragments of DRD2 promoter were amplified from pig genomic DNA using the primers (Supplementary Table S1) and inserted into the MIu I/Hind III sites of pGL3-Basic plasmid. The mutants of transcription factor binding sites were generated using a Trelief^™^ SoSoo Cloning Kit (Tsingke Biotechnology) and mutagenic primers (Supplementary Table S1). The overexpression plasmids of IRF1 and IRF2 were synthesized by Tsingke Biotechnology. All plasmids were sequenced to confirm the correct insertion.

### 2.5. Cell Culture, Plasmid Transfection, and Luciferase Reporter Assay

Porcine kidney-15 (PK15) (ATCC^®^ACS-4004^™^) and 293T (ATCC^®^ACS-4004^™^) cells were cultured in high-glucose medium with 10% fetal bovine serum (FBS) (10%FBS + 90%DMEM) (FBS, Gibco) (DMEM/High, Gibco) at 37 °C and 5% CO_2_. To isolate and culture porcine neuronal cells (PNCs) briefly, the porcine brain tissue was obtained from a newborn piglet, then the piglet was anesthetized by ethyl ether and euthanized. Dissected brain tissue was then cut into small pieces (about 1 mm^3^), and was digested with Papain (Biosharp, Hefei, China) for 30 min. Then, the cells were seeded with high-glucose medium (20%FBS + 80%DMEM). After 48 h, the cells were cultured with Dulbecco’s minimum essential medium/nutrient F-12 (DMEM/F-12, Gibco) supplied with 15% FBS at 37 °C with 5% CO_2_. When the number of cells grew by approximately 70~80%, they were transfected with plasmid DNA or small interfering RNAs (siRNA) utilizing Lipofectamine 2000 (Invitrogen, Shanghai, China). After 24 h, the 293T and PK15 cells were collected using luciferase assay buffer (Promega, Madison, WI, USA). The luciferase activity of cell lysates was assayed by Promega dual luciferase assay system. Porcine nerve cells were transfected with plasmid DNA or small interfering RNAs (siRNA) utilizing Lipofectamine 3000 (Invitrogen, Shanghai, China). All transfection/silencing experiments were repeated three times.

### 2.6. RNA Isolation and RT-qPCR

Total RNA was isolated from transfected cells and different tissues (muscle, liver, lung, heart, pituitarium, cerebellum, cerebrum, and hypothalamus) of the Suhuai pigs, using TRIzol (Invitrogen, Carlsbad, CA, USA) according to the manufacturer’s instructions [36]. The purity of RNA was determined with a NanoPhotometer^®^ Spectrophotometer (IMPLEN, CA, USA) at 260/280 nm. Total RNA was reversely transcribed into cDNA with the HiScript III RT SuperMix (Vazyme Biotech, Nanjing, China). Removal of residual genomic DNA and synthesis of cDNA were performed with the HiScript III RT SuperMix (Vazyme Biotech). RT-qPCR was performed on a QuanuStudio 5 using SYBR Green Master Mix (Vazyme Biotech). Relative expression levels were calculated using the 2−ΔΔCt method [37]. Gene expression levels were normalized to the expression level of glyceraldehyde-3-phosphate dehydrogenase (GAPDH). RT-qPCR was repeated three times, and primers are shown in Supplementary Table S2.

### 2.7. Western Blotting

Cell protein lysates were collected with radio immunoprecipitation assay (RIPA) buffer (Beyotime Biotechnology, Nanjing, China) and 1% protease inhibitors (PMSF) (*v/v*) (Biosharp, China). Total protein extracts were separated using 4% to 20% sodium dodecyl sulfate polyacrylamide gel electrophoresis (SDS–PAGE) gels (Genscript, Biotechnology, Piscataway, NJ, USA) and blotted onto polyvinylidene fluoride (PVDF) (Millipore, USA). The membranes were blocked with 5% bovine albumin (BSA) (Beyotime Biotechnology, China) at 4 °C for 2 h, then incubated overnight with the following primary antibodies: immunoreactive proteins detected with rabbit polyclonal antibody to DRD2 (1: 2000; Affinity), IRF1 (1: 2000; Affinity), IRF2 (1: 2000; Affinity), and rabbit polyclonal antibody to GAPDH (1: 4000; Affinity). The suitable secondary antibody used was anti-rabbit (1: 8000; Affinity), and chemiluminescence was detected by Image LAS-4000 system. The band density was analyzed by ImageJ 1.53c software.

### 2.8. Chromatin Immunoprecipitation (ChIP) Assay

ChIP assays were performed as previously described [8]. Cells were briefly crosslinked with 1% formaldehyde at room temperature for 10 min. The reaction was quenched with 125 mM glycine. Cells were collected, washed twice with cold phosphate-buffered saline (PBS), and resuspended in Lysis Solution Ⅰ (10 mM HEPES, 0.2%NP-40, 10 mM NaCl, 1 mM EDTA, and 1xprotein inhibitor) and Lysis Solution Ⅱ (SDS, 10 mM EDTA, 10 mM Tris-HCl, and 1xprotein inhibitor). Nuclear extracts were sonicated to generate chromatin fragments with an average size of 300~500 bp on a Q800R sonicator (QSonica). Then, chromatin immunoprecipitation experiment was carried out using a ChIP assay kit (Abcam, ab117138-ChIP Kit-One Step) according to the manufacturer’s instructions.

### 2.9. Immunofluorescence Assay

PNCs were seeded on coverslips and cultured routinely. After 72 h, when the cells reached 70% confluence, they were fixed for 15 min in 4% paraformaldehyde (Beyotime, Shanghai, China). The cells were washed with PBS three times, incubated with 0.1 mL Triton X-100 (0.5%) for 20 min, and blocked for 1 h with QuickBlock^™^ blocking buffer (Beyotime, P0260). Then, the cells were incubated with one of the following primary antibodies: anti-microtubule-associated protein 2 (anti-MAP2) (1: 100; Affinity); or anti-tubulin (anti-TUJ1) (1: 100; Affinity), at room temperature for 1 h, washed with PBS, and incubated with fluorescein-conjugated goat anti-rabbit secondary antibodies (1: 500; Proteintech) for 1 h. Nuclei were stained using 4,6-diamidino-2-phenylindole (DAPI) (GeneCopoeia). Using a confocal laser microscope (Thermo; 200,960s), randomly-selected fields were evaluated for each slide for the strength of the staining signals. The number of positive pixels in each field is an indicator of the strength of the staining signal and level of gene expression.

### 2.10. Flow Cytometry

The level of cell apoptosis was assessed by flow cytometry with an Annexin V-fluorescein isothiocyanate (FITC)/propidium iodide (PI) apoptosis detection kit (Vazyme, A211, Nanjing, China) according to the manufacturer’s protocol. In brief, cells were seeded in a six-well plate and transfected with a negative control or siDRD2 or overexpression plasmids and then incubated for 48 h. Afterwards, cultured cells were harvested by trypsinization and cells were washed twice with PBS and resuspended in Annexin V-binding buffer. Cell suspension was then incubated with 5 μL of Annexin V (AV)-FITC and 5 μL of PI staining solution in the dark for 10 min, and the cells were read by a FACS can flow cytometer. A total of 10,000 cells were detected, and to calculate the apoptosis rate, FlowJo v7.6 software (Stanford University, Stanford, CA, USA) was used to analyze the data.

### 2.11. Statistical Analysis

Data analysis was performed using SAS Studio (SAS Institute Inc. Cary, NC, USA). Chi-square tests were used to compare the difference of the gene and genotype frequencies of the pigs between the most aggressive and the least aggressive pigs. The GLIMMIX procedures with a model option DIST = EXPO in SAS were used to analyze the differences of behavioral indicators of 36 h after mixing. Genotype, sex, and group were used as the fixed effects to split the pigs into two equal-sized groups of the two least aggressive and two most aggressive pigs in each pen of 9 or 10 pigs, and the random effect was the pen. For the cell experiments, statistical significance was assessed using the student’s *t*-test. Data were reported as means ± standard error and *p*-value < 0.05 was considered significant.

## 3. Results

### 3.1. Identification of the Core Promoter Region of the Porcine DRD2 Gene

The sequence (2250 bp) on the upstream of the porcine DRD2 gene was used for the prediction of the promoter, transcription factor binding sites, and CpG islands. Two transcription initiation sites, seven promoter regions (from −1818 bp to −1517 bp) (Supplementary Tables S3 and S4), and four CpG islands (from −1894 bp to −1388 bp, −1376 bp to −956 bp, −859 bp to −754 bp, and −653 bp to −539 bp) (Supplementary Table S5) were predicted for the porcine DRD2 gene.

**Figure 1:**
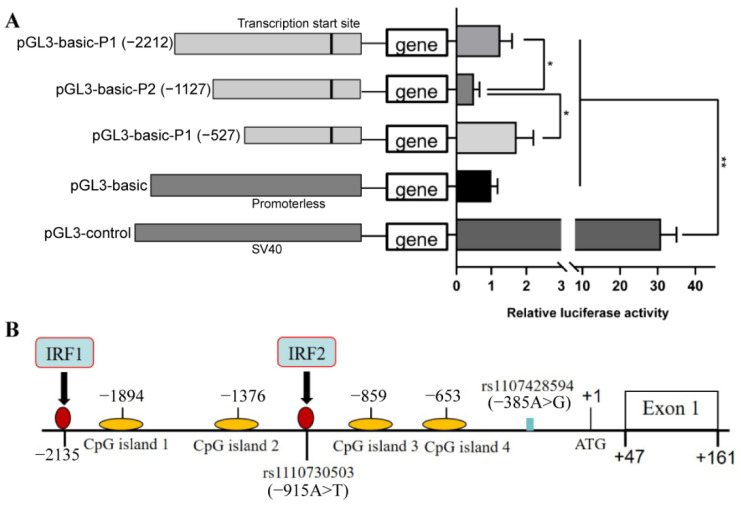
Deletion analysis of the dopamine receptor D2 (DRD2) promoter. (**A**) A series of progressive deletion mutants’ promoter activities analysis by luciferase activity assay: (left) the mutants constructed into pGL3-basic luciferase reporter vector; (right) the relative promoter activity of deletion mutants. The pGL3-basic vector was used as a negative control, the pGL3-control vector was used as a positive control, and the pGMLR-TK luciferase reporter vector was applied as an internal control. (**B**) Schematic diagram of the interferon regulatory factor (IRF) 1 or IRF2 binding site (arrow, solid red circle) in the DRD2 promoter. CpG islands were indicated by orange ellipses and the nucleotides were numbered relative to it. Data were presented as means ± standard errors (SE) of three replicates. * *p* < 0.05, ** *p* < 0.01.

To identify the core promoter region, three deletion fragments of the DRD2 gene promoter were constructed into pGL3-Basic luciferase reporter vectors based on the predicted promoter regions. The pGL3-basic empty vector and the pGL3-control vector were used as the negative control group and positive control group, respectively. The luciferase activity of vector P1 (−2212 to −1127 bp) and P3 (−527 to +47 bp) in the 5′-flanking region was significantly greater than that of vector P2 (−1127 to −527 bp) (*p* < 0.05) in 293T cells. No difference was found in the luciferase activity between vector P1 and P3 (*p* > 0.05) (Figure 1A). These results suggest that the 5′-flanking region from −2212 to −1127 bp is a transcriptional promotion region, but the 5′-flanking region from −1127 to −527 bp is a transcriptional suppression region.

To explore whether there are transcription factors binding in the transcriptional promotion region or the transcriptional suppression region, we predicted a potential site (TTTCC) for IRF1 in the transcriptional promotion region and a potential site (AAGTGA) for IRF2 in the transcription suppression region (Figure 1B).

### 3.2. Identification of Functional SNPs Related to Aggressive Behavior in the Upstream Region of Porcine DRD2 Gene

**Figure 2:**
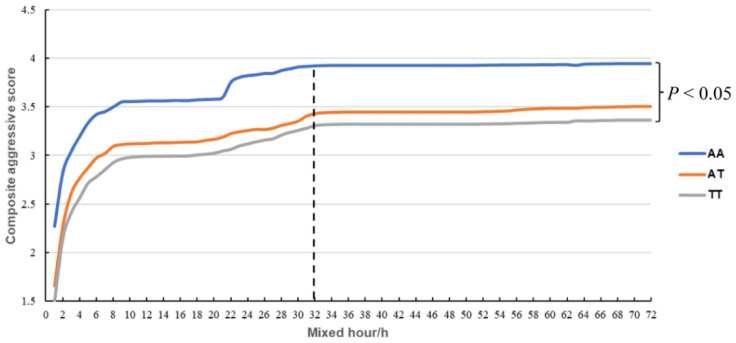
The curve of composite aggressive score (CAS) of pigs with different genotype (SNP rs1110730503) during the first 72 h after mixing.

There are two SNPs, rs1110730503 (−915A > T) and rs1107428594 (−385A > G), in the 5′-UTR of the DRD2 gene in Suhuai pigs (Table 1). The three genotypes for rs1110730503 (−915A > T) were significantly different for the duration of active attack, the duration of bullying, and the composite aggressive score (CAS) (*p* < 0.05) (Table 2). The AA genotype had a significantly greater composite aggressive score (CAS) than the TT genotype (*p* < 0.05) (Table 2) (Figure 2). The CAS was gradually increased within 32 h after mixing, and then stabilized (Figure 2). These data indicated that AA-genotype pigs have greater aggressive behavior than TT-genotype pigs. In addition, the three genotypes for rs1107428594 (−385A > G) were significantly different for the duration of standoff (*p* < 0.05), as the AA genotype was significantly greater than the GG genotype (Table 2).

### 3.3. Transcription Factor IRF1 Binds to the DRD2 Promoter

**Figure 3:**
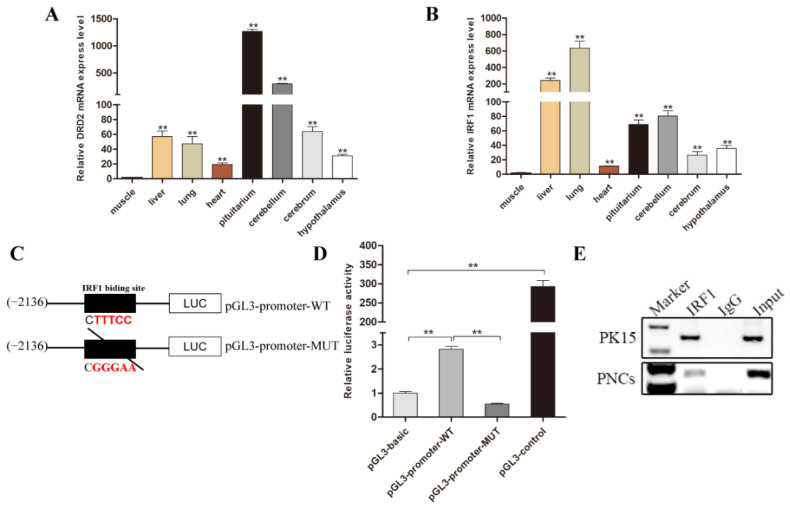
Expression profile of porcine DRD2 and IRF1 gene, and site-directed mutagenesis of IRF1 binding site in the DRD2 promoter. (**A**,**B**) Expression characteristics of porcine DRD2 and IRF1 gene at different tissues. (**C**) Site-directed mutation schematic diagram of the predicted IRF1 binding site in the DRD2 promoter. (**D**) Site-directed mutation of IRF1 binding site of DRD2 gene by luciferase activity assay. Wild-type (WT) or mutant (MUT) of the DRD2 promoter were transfected into 293T cells, respectively. The pGMLR-TK luciferase reporter vector was applied as an internal control, and the pGL3-Basic vector was used as a negative control, the pGL3-control vector was used as a positive control. (**E**) Binding of IRF1 on the DRD2 promoter was demonstrated using ChIP assays in porcine kidney-15 (PK15) and neuronal cells (PNCs). After immunoprecipitation, identified the IRF1 binding site by polymerase chain reaction (PCR) amplification. Input was total fragmented DNA. Precipitated chromatin with normal Immunoglobulin G (IgG) was applied as the negative control. Data were presented as means ± standard errors (SE) of three replicates. ** *p* < 0.01. The uncropped western blot figures can be accessed in Supplementary files.

The tissue expression profile indicated that both DRD2 and IRF1 were extensively expressed in various tissues of pigs. The expression level of the DRD2 gene was highest in brain tissues (Figure 3A), while the transcription factor IRF1 was highly expressed in the lung and liver, and moderately expressed in brain tissues, including the cerebellum, pituitary, and hypothalamus (Figure 3B). To determine the function of IRF1 binding sites, site-directed mutagenesis was conducted to mutate the IRF1 transcription factor binding site in pGL3-promoter plasmid using a WT pGL3-promoter construct as a template (Figure 3C). The relative luciferase activity of the pGL3-promoter-WT group was greater than that of the pGL3-promoter-MUT group (*p* < 0.05) (Figure 3D).

To further verify the binding of transcription factor IRF1 with the promoter region of DRD2 gene, we performed a chromatin immunoprecipitation (ChIP) assay using porcine kidney-15 (PK15) and neuronal cells. Sheared crosslinked DNA was immunoprecipitated by a specific anti-IRF1 or anti-Immunoglobulin G (anti-IgG). Precipitated DNA was purified and used as a template for PCR amplification. In addition, the specific PCR primers (Supplementary Table S1) were contained in the IRF1 binding sites sequence. The ChIP analysis showed that a band was observed in both the anti-IRF1 and Input chromatin lanes in the PK15 and PNCs group (Figure 3E). In contrast, no specific band was observed in the rabbit anti-IgG group. Our ChIP assay demonstrated the specific interaction between transcription factor IRF1 and the promoter region of the DRD2 gene.

### 3.4. Transcription Factor IRF1 Upregulated the Expression Level of Porcine DRD2 Gene by Binding to Its Promoter Region

**Figure 4:**
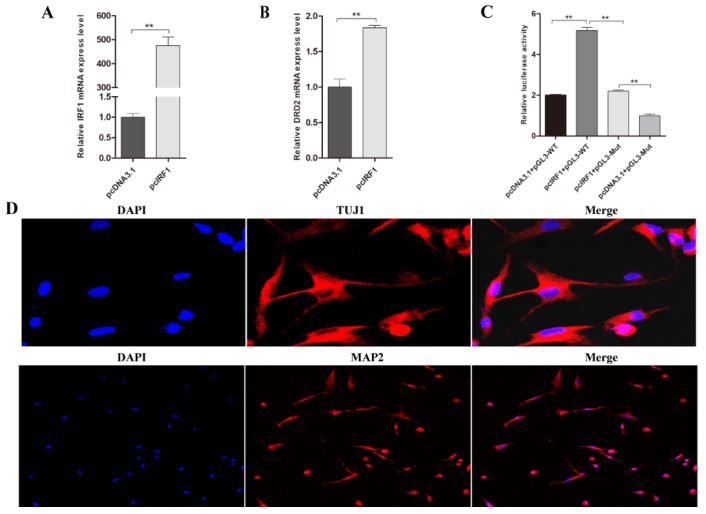

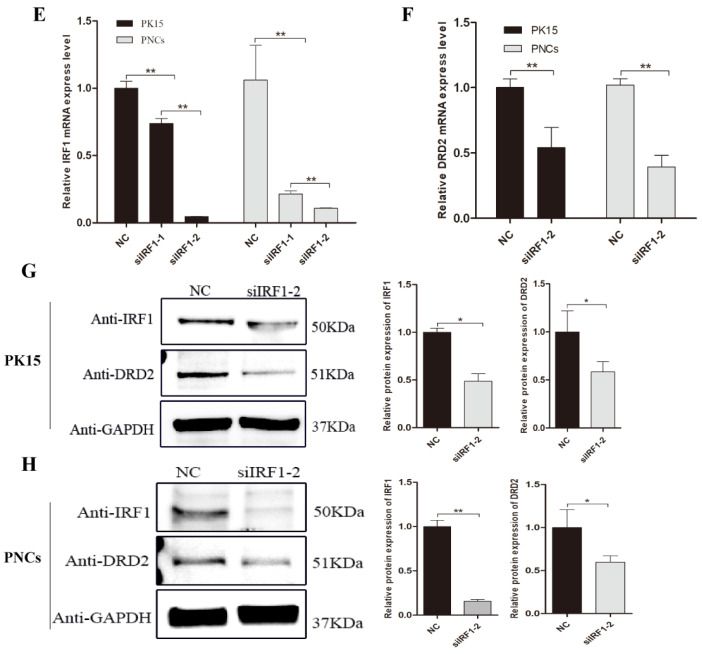
Transcription factor IRF1 promoted the transcription activity of DRD2 gene. (**A**) The IRF1 mRNA expression in PK15 cells which transfected with pcIRF1 or pcDNA3.1(+). The mRNA levels were normalized to GAPDH. (**B**) DRD2 mRNA expression in PK15 cells which transfected with pcIRF1 or pcDNA3.1(+). The mRNA levels were normalized using GAPDH. (**C**) Analysis of the binding region of IRF1; pcIRF1 and pGL3-promoter-WT or pGL3-promoter-MUT vectors were co-transfected into PK15 cells. The pcDNA3.1 vector was applied as a vector control. (**D**) Immunofluorescence staining of MAP2 or TUJ1 expression in the PNCs. Nuclei were stained using DAPI. (**E**,**F**) RT-qPCR analyzed the IRF1 knockdown efficiency and DRD2 gene mRNA expression level. PK15 and PNCs were transfected with two different siRNAs and negative control (NC) (2.5 μL) by lip3000 for 24 h. The mRNA levels were normalized using GAPDH. (**G**,**H**) The IRF1 knockdown efficiency and the protein level of the DRD2 gene were determined by western blotting analysis. PK15 and PNCs were infected with the siRNA and negative control (NC) (5 μL) by lip3000 for 48 h. Data were presented as mean ± SE of three replicates. * *p* < 0.05, ** *p* < 0.01. The uncropped western blot figures can be accessed in Supplementary files.

To explore the effects of IRF1 binding to the promoter region of the DRD2 gene, we constructed an IRF1 overexpression vector. The cDNA of the porcine IRF1 gene was inserted into the eukaryotic expression vector. Then, the pcIRF1 overexpression vector and pGL3-promoter-WT vector or pcDNA3.1 empty vector and pGL3-promoter-MUT vector were co-transfected into PK15 cells to detect the luciferase activity and the mRNA expression level of the porcine IRF1 and DRD2 genes by RT-qPCR. The results demonstrated that with an increased level of expression for porcine IRF1, the expression level of DRD2 also increased (Figure 4A,B). Meanwhile, the relative luciferase activity of the pGL3-promoter-WT and the pcIRF1 vector co-transfected group was greater than that of the pGL3-promoter-MUT and the pcIRF1 vector co-transfected group (*p* < 0.05) (Figure 4C), which demonstrated that transcription factor IRF1 upregulated the expression level of the porcine DRD2 gene.

A previous study found that the DRD2 gene not only plays a role in the classic DA pathway but is also essential for signal transduction in the brain [38]. Our tissue expression profile indicated that both DRD2 and IRF1 are extensively expressed in brain tissues of pigs. Therefore, we isolated and cultured porcine neuronal cells (PNCs) from porcine brain tissues. We identified PNCs using two antibodies: anti-microtubule-associated protein 2 (Anti-MAP2) and anti-tubulin (Anti-TUJ1), which were specifically used to identify neuronal cells by immunofluorescence assay. The cells’ bodies and the short nervous processes can be clearly observed in subtotal neurons (Figure 4D), which suggests that we successfully isolated PNCs.

To verify the effects of IRF1 knockdown on the expression level of the porcine DRD2 gene, small interfering RNAs (siRNAs) were used to knock down the expression level of IRF1 by transfecting siRNAs to PK15 cells and PNCs. The results indicated that the relative mRNA expression levels of siIRF1-2 were lower than those of siIRF1-1 (*p* < 0.01) in PK15 cells and PNCs (Figure 4E). Thus, siIRF1-2 was transfected into PK15 cells and PNCs to detect the mRNA and protein expression level of the DRD2 gene. RT-qPCR (Figure 4F) and western blotting showed that IRF1 knockdown significantly reduced (*p* < 0.05) the mRNA and protein expression level of the porcine DRD2 gene both in PK15 cells (Figure 4G) and PNCs (Figure 4H). These results demonstrated that the binding of transcription factor IRF1 with the promoter region of the porcine DRD2 gene upregulated the mRNA and protein expression level of the DRD2 gene.

### 3.5. Transcription Factor IRF2 Downregulated the Expression Level of Luciferase Gene by Binding to the Promoter Region

**Figure 5:**
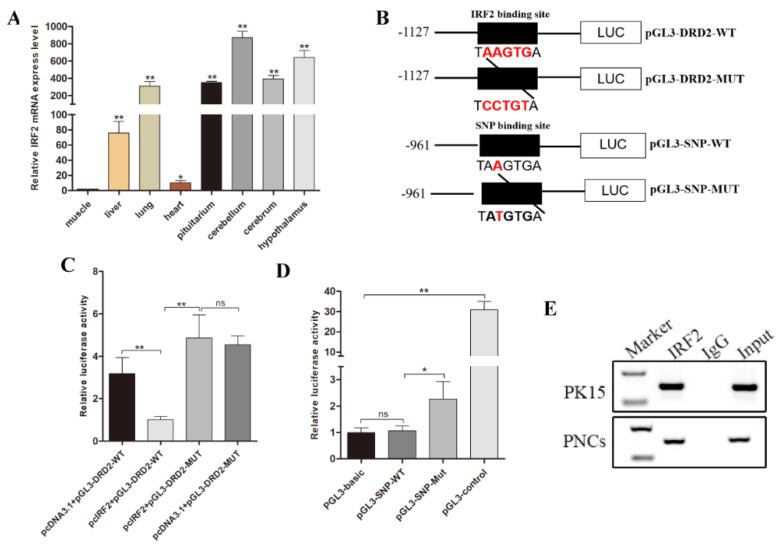
Expression profile of porcine IRF2 gene and site-directed mutagenesis of IRF2 binding site in the DRD2 transcriptional suppression region. (**A**) Expression characteristics of porcine IRF2 gene at different tissues by RT-qPCR analysis. (**B**) Site-directed mutation schematic diagram of the predicted IRF2 binding site in the DRD2 promoter. (**C**) Site-directed mutation of IRF2 binding site of DRD2 gene by luciferase activity assay. Wild-type or mutant of the DRD2 promoter luciferase reporter vectors were transfected into 293T cells, respectively. The pGL3-Basic vector was used as a negative control, and the pGL3-control vector was used as a positive control. (**D**) Luciferase reporter gene assays of porcine DRD2 alleles containing rs1110730503 (−915A/T). Two DRD2 genotype luciferase reporter vectors were constructed and transfected into 293T cells. The pGL3-Basic vector was used as a negative control, and the pGL3-control vector was used as a positive control. (**E**) The IRF2 binding sits on the DRD2 gene were identified using ChIP assays in PK15 and PNCs. After immunoprecipitation, the IRF2 binding sites were demonstrated by PCR amplification. Input was total fragmented DNA. Precipitated chromatin with normal IgG was applied as the negative control. Data were presented as mean ± SE. of three replicates. * *p* < 0.05, ** *p* < 0.01. The uncropped western blot figures can be accessed in Supplementary files.

The tissue expression profiles indicated that the transcription factor IRF2 was highly expressed in the brain tissues, including the cerebellum, hypothalamus, cerebrum, and pituitary (Figure 5A). We predicted the binding sites of transcription factor IRF2 to the transcription repression region of the DRD2 gene. Interestingly, we also found that SNP rs1110730503 (−915A/T), associated with aggressive behavior, is located in the binding sites of IRF2. To identify whether this SNP influences the binding of IRF2 to the promoter of the porcine DRD2 gene, we constructed luciferase reporter vectors containing the IRF2 binding site sequence (TAAGTGA) (pGL3-DRD2-WT), the mutant IRF2 binding site sequence (TCCTGTA) (pGL3-DRD2-MUT), the IRF2 binding site sequence with wild type allele −915A (TAAGTGA) (pGL3-SNP-WT), or the IRF2 binding site sequence with mutant allele −915T (TATGTGA) (pGL3-SNP-MUT) (Figure 5B). In the 293T cells, the relative luciferase activity of the pGL3-DRD2-MUT group was greater than that of the pGL3-DRD2-WT group (*p* < 0.05) (Figure 5C). Furthermore, the relative luciferase activity of the pGL3-SNP-MUT group was greater than that of the pGL3-SNP-WT group (*p* < 0.05) (Figure 5D). In addition, our ChIP assay in porcine PK15 and PNCs identified the interaction between IRF2 and DRD2 protein. A band was detected in the input and antibody anti-IRF2 treatments (Figure 5E). These results indicate that transcription factor IRF2 downregulates the expression level of the luciferase gene by binding to its promoter region, and the SNP rs1110730503 (−915A/T) as a potential regulation element influences the binding effect of IRF2 to the promoter region of the luciferase gene.

### 3.6. IRF2 as a Transcription Repressor Downregulated the Expression Level of Porcine DRD2 Gene

**Figure 6:**
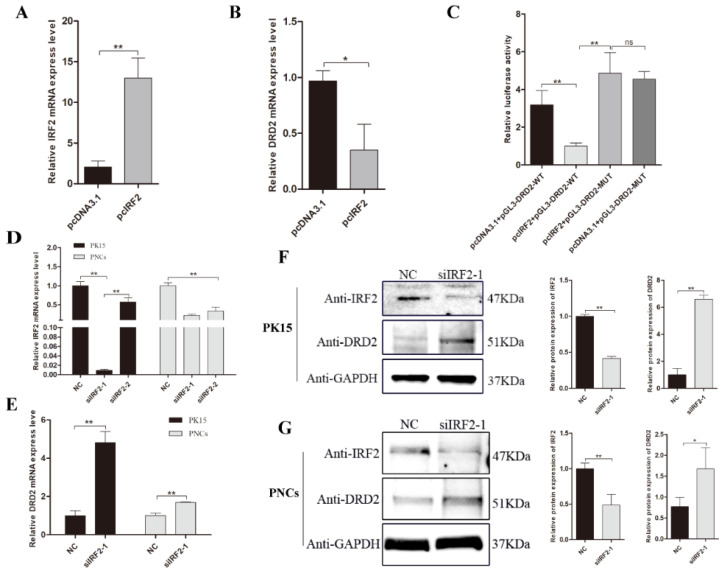
IRF2 as a transcription repressor downregulated the DRD2 gene expression. (**A**,**B**) IRF2 mRNA expression in PK15 cells which transfected with pcIRF2 or pcDNA3.1(+). The mRNA levels were normalized using GAPDH. IRF2 overexpression diminished DRD2 transcription in PK15 cells. (**C**) Analysis of the binding region of IRF2 when co-transfected pcIRF2 and pGL3-DRD2-WT or pGL3-DRD2-MUT vectors into PK15 cells; the pcDNA3.1 vector was applied as a vector control. (**D**,**E**) The IRF2 knockdown efficiency and the expression of DRD2 gene were detected by RT-qPCR analysis. PK15 and PNCs were infected with two different IRF2 siRNAs and negative control (NC) (2.5 μL) by lip3000 for 24 h. The mRNA levels were normalized to GAPDH. (**F**,**G**) IRF2 knockdown efficiency and the protein level of DRD2 gene were detected by western blotting analysis. PK15 and PNCs were infected with the siRNA and negative control (NC) (5 μL) by lip3000 for 48 h. Data were presented as mean ± SE of three replicates. * *p* < 0.05, ** *p* < 0.01. The uncropped western blot figures can be accessed in Supplementary files.

Next, we investigated the effects of IRF2 on the expression level of the porcine DRD2 gene. The whole cDNA sequence of porcine IRF2 gene was inserted into eukaryotic expression vector pcDNA3.1 to generate the IRF2 overexpression vector (pcIRF2). As expected, when IRF2 was overexpressed (Figure 6A), the relative mRNA expression level of the DRD2 gene was decreased in porcine PK15 cells (*p* < 0.05) (Figure 6B). Meanwhile, when the IRF2 overexpression vector and pGL3-DRD2-WT vector were co-transfected into PK15 cells, the relative luciferase activity was significantly decreased compared to the other treatments (*p* < 0.05) (Figure 6C).

A siRNA-mediated knockdown of IRF2 in PK15 cells and PNCs showed that siRNA siIRF2-1 had a better efficiency of inhibition in PK15 cells and PNCs (*p* < 0.01) (Figure 6D). Thus, we transfected the siRNA siIRF2-1 into PK15 and PNCs to detect the mRNA and protein expression of the DRD2 gene. The inhibition of IRF2 resulted in greatly increased mRNA expression of the DRD2 gene in PK15 and PNCs (*p* < 0.01) (Figure 6E). Consistent with these results, western blotting assays indicated that as the inhibition of IRF2 was induced, the DRD2 protein expression level was increased (*p* < 0.01) both in PK15 (Figure 6F) and PNCs (Figure 6G).

### 3.7. Transcription Factor IRF1 and IRF2 Are Functionally Antagonistic to Each Other in PNCs

**Figure 7:**
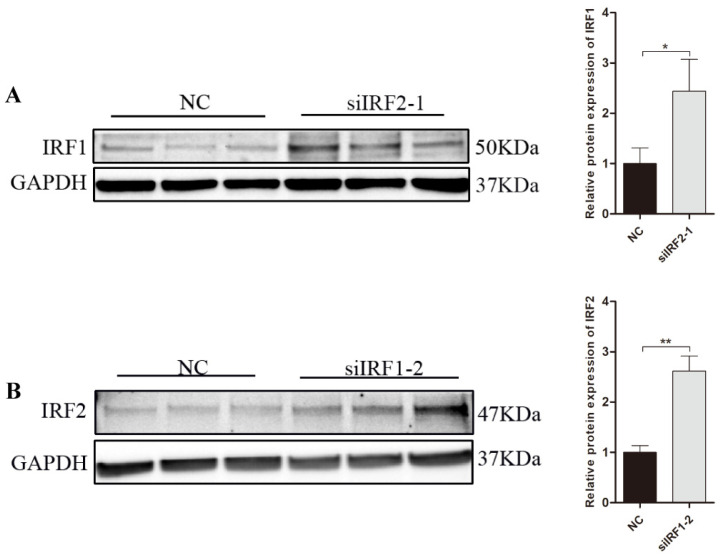
Transcription factors IRF1 and IRF2 are functionally antagonistic to each other. (**A**) The relative protein levels of IRF2 were detected by western blotting analysis while siIRF1-2 transfected into PNCs for 48 h. (**B**) Western blotting analysis of IRF1 protein expression in PNCs while transfected with siIRF2-2 and negative control (NC). The protein levels were normalized to GAPDH. Data are presented as mean ± SE. of three replicates. * *p* < 0.05, ** *p* < 0.01. The uncropped western blot figures can be accessed in Supplementary files.

To investigate the relationship between transcription factor IRF1 and IRF2 in PNCs, we detected the protein expression of IRF1 when IRF2 was inhibited (Figure 7A), as well as the protein expression of IRF2 when IRF1 was inhibited (Figure 7B) by western blotting. The results indicated that IRF1 and IRF2 were functionally antagonists to each other in PNCs (*p* < 0.05).

### 3.8. Effect of DRD2 Gene on Apoptosis of Porcine Neuroglial Cells

**Figure 8:**
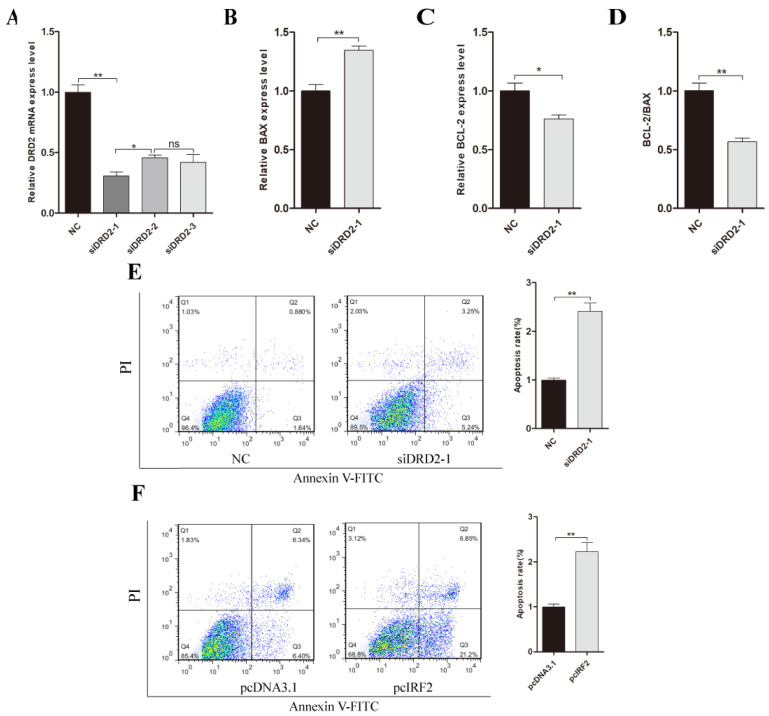
Effect of inhibition of DRD2 gene on apoptosis of porcine neuroglial cells. (**A**) The interference efficiency of three siRNA of DRD2 gene in porcine neuroglial cells. (**B**,**C**) RT-qPCR analysis of BAX and BCL-2 mRNA expression in porcine neuroglial cells which transfected with siDRD2-1 or negative control. The mRNA levels were normalized using GAPDH. (**D**) BCL-2/BAX ratio. (**E**,**F**) FACS analyses of the apoptosis rates of porcine neuroglial cells after being transfected with siDRD2-1 or NC and pcDNA3.1 or pcIRF2. Data are presented as mean ± SE of three replicates. * *p* < 0.05, ** *p* < 0.01.

The DRD2 gene is related to the immunity system in neuroglial cells [39]. To investigate the effect of DRD2 on apoptosis of neuroglial cells, we used three DRD2 small interfering RNAs to transfect into neuroglial cells. The results indicated that siDRD2-1 had greater interference efficiency compared with siDRD2-2 and siDRD2-3 (Figure 8A) (*p* < 0.05). RT-qPCR of BAX and BCL-2 genes indicated that the siDRD2-1 group resulted in lower mRNA expression of BAX but greater expression of BCL-2 compared with the negative control group (Figure 8B,C) (*p* < 0.05); the ratio of BCL-2/BAX was significantly lower in the si*DRD2*-1 group (Figure 8D) (*p* < 0.05). These results indicated that the DRD2 gene inhibits porcine neuroglial cell apoptosis. A fluorescence activated cell sorting (FACS) analysis also revealed that the neuroglial cells both in siDRD2-1 and pcIRF2 group had a higher (*p* < 0.05) apoptosis rate than the negative control or pcDNA3.1 group (Figure 8E,F).

## 4. Discussion

DRD2 was found to be related to aggressive behavior in chickens [40]. In our present study, four CpG islands were predicted in the upstream region of the porcine DRD2 gene. A previous study reported that DNA methylation regulates gene expression level by changing the chromatin structure, DNA stability, and the way in which DNA interacts with proteins [41]. Meanwhile, DRD2 methylation was positively associated with robust activation in the striatum in response to reward cues [42]. Whether these predicted CpG islands are involved in regulation of DRD2 expression remains to be further studied.

Neuroscience has now revealed a core network of the hypothalamus [43], prefrontal cortex (PFC) [44], and dorsal raphé nucleus (DRN) [45] regions of the brain, which are essential for the production of aggressive behavior [46]. Previous studies have shown that pulvinar is mutually and extensively connected with the prefrontal cortex, sensory cortex, superior colliculus, and amygdala [47] and plays very important roles in contextual multi-sensory processing and emotional response [48,49,50]. The dysfunction of pulvinar has been reported to be associated with cognitive and emotional deficits in depression, including aggression [51,52]. In our research, we successfully isolated and cultured the porcine neuronal cells to explore the functional mechanism of the DRD2 gene. Further in-depth research between brain core network and the porcine DRD2 gene on aggressive behavior is needed.

The promoter is a key component of genes, controlling the initiation location and expression abundance of the gene [53]. We demonstrated that the core promoter region of DRD2 gene is located in a transcription activator binding region from −2212 to −1127 bp. With a deletion fragment in this region, the transcription activity of plasmid was significantly greater. At the same time, a transcription factor binding was predicted in this region. Transcription activators are a group of proteins that bind to specific consensus sequences (cis elements) in the promoter region to upregulate gene expression [54]. Consistent with previous studies [25,26], our present study demonstrated a functional interaction between IRF1 and the DRD2 gene. As a target gene of IRF1, the mRNA and protein expression level of the DRD2 gene were increased by the binding of IRF1 into its promoter in PNCs.

In the present study, we found that the luciferase activity of plasmid with a deletion fragment P2(−1127~−527 bp) of the DRD2 promoter significantly decreased, while transcription factor IRF2 was predicted to bind into this region. Transcription repressors are a group of proteins that play a crucial role in the negative regulation of gene transcription [55]. Here, we provided supporting evidence that IRF2 as a transcription repressor downregulated DRD2 gene expression. We observed that the over-expression of IRF2 decreased the expression level of the DRD2 gene, while the inhibition of IRF2 increased its expression in PNCs.

Several previous studies have investigated genetic variants associated with aggressive behavior [56,57]. In the study on the relationship between the dopamine system and aggressive behavior in children, the CC genotype of rs1079598 in the DRD2 gene was overrepresented in aggressive children compared to controls [58]. Similar to this study, we also identified a functional SNP rs1110730503 (A > T) which is associated with aggressive behavior. In addition, this SNP is located in IRF2 binding sites, which suggests a new layer of genetic and functional mechanisms of the SNP. Previous studies have demonstrated the important roles of SNPs in regulation of gene expression [7]. For example, SNP −287T/C in the promoter region of the tissue factor pathway inhibitor (TFPI) gene exerted differential impacts on mRNA expression level of the TFPI gene [59]. In our present study, we found that the A allele of rs1110730503 had lower transcription activity than that of the T allele because of the binding of IRF2.

In previous study, researchers found that aggression is often comorbid with neuropsychiatric diseases such as drug addiction [60], depression [61], and Parkinson’s disease (PD) [62]. Furthermore, they found that the learned aggression was characterized by low DRD2 levels [63], and the expression level of DRD2 was decreased in the brain tissue of PD mice, which suggested that nardosinone may reduce the motor and cognitive symptoms in the animal PD model by regulating DRD2 expression [62]. In addition, mice with depression also had significantly lower DRD2 expression compared with control mice. The DRD2 agonist Ropinirole (ROPI) can be used as an antidepressant drug for the treatment of depressive disorders [19]. These findings indicate that low DRD2 level is related to aggressive behavior. Meanwhile, in our previous study of blood-based biomarkers associated with aggression in weaned pigs after mixing, we found that aggressive pigs had less serotonin and dopamine at 24 h after mixing [30]. Our present study found that IRF2 binds to the DRD2 promoter with the A allele of rs1110730503, resulting in a decrease in the transcription activity and the expression level of the DRD2 gene. We also found that the AA-genotype pigs were more aggressive than those which were TT-genotype, which is consistent with previous studies.

Transcription factors IRF1 and IRF2 were originally identified as transcriptional regulators of the interferon (IFN) and IFN-stimulated genes [64]. They also modulate immune response and play a role in regulation of cell growth [65]. Interestingly, we found that IRF1 and IRF2 were functionally antagonistic to each other in PNCs, which is consistent with previous studies [26,66].

In the central nervous system, DA exerts a multitude of functions, including control of locomotion [38], affective behavior, and emotions [67]. Furthermore, DRD2 as a specific receptor of DA was not only involved in reward processing, emotion regulation, and decision making [68], but also participated in the regulation of innate immunity and suppression of neuroinflammation [69]. A previous study showed that DRD2 knockout mice showed significant inflammatory response in multiple central nervous system regions in neuroglial cells [39]. In our present study, a knocked-down DRD2 gene and overexpressed IRF2 both induced the apoptosis rate in porcine neuroglial cells. These findings suggest that the porcine DRD2 gene might influence the aggressive behavior through immune pathways in the brain. Further studies will be needed to address the hypothesis.

## 5. Conclusions

**Figure 9:**
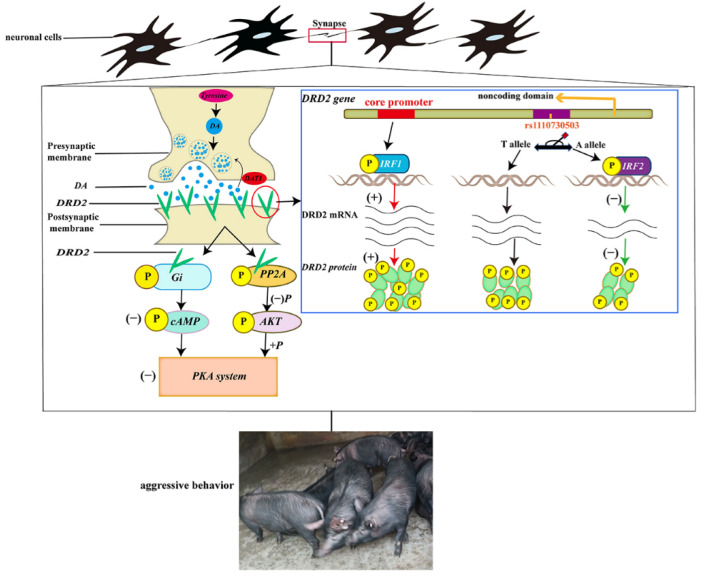
Molecular mechanism of functionally antagonistic transcription factors IRF1 and IRF2 regulating the transcription of the DRD2 gene associated with aggressive behavior of weaned pigs.

The promoter activity analyses indicate that the core promoter region of the porcine DRD2 gene is located between −2212 bp and −1127 bp from the initiation site of transcription. Meanwhile, two SNPs in the promoter region of the DRD2 gene were associated with aggressive behavior of weaned pigs after mixing. We also discovered that transcription factors IRF1 and IRF2 are crucial regulatory factors for DRD2 transcription, and identified the binding sites of IRF1 and IRF2 on the promoter region of the DRD2 gene. An SNP (rs1110730503) in the binding site of IRF2 affected the binding between IRF2 and the promoter of the DRD2 gene, regulating the mRNA and protein expression level of the DRD2 gene, which could be the reason for changes in aggressive porcine behavior (Figure 9).

## Figures and Tables

**Table 1:** Genetic polymorphism analysis of porcine DRD2 gene promoter (−2250 bp).

Mutation Site Number	Mutation Site Position	Serial Number	Alleles	Genotype
1	41134564 5′-UTR	rs1110730503	A > T	AA/AT/TT
2	41134034 5′-UTR	rs1107428594	A > G	AA/AG/GG

**Table 2:** Aggressive behavior indicators of pigs with different genotypes of SNP rs1110730503 and rs1107428594 in porcine DRD2 gene.

SNP	Genotype	Number	Composite Aggressive Score (CAS)	Duration of Active Attack(s)	Duration of Bullying	Duration of Standoff(s)	Standoff Frequency
rs1110730503 (41134564)	AA	26	3.9295 ± 0.1984 a	6.0369 ± 0.2 a	2.9482 ± 0.1978	2.7236 ± 0.2091	6.6762 ± 0.2013
	AT	49	3.4457 ± 0.1501 b	5.5254 ± 0.1514 b	2.9498 ± 0.1487	2.3509 ± 0.1462	6.2924 ± 0.1461
	TT	107	3.3232 ± 0.105 b	5.35 ± 0.1038 b	2.9183 ± 0.1029	2.2571 ± 0.1087	6.5448 ± 0.1063
	*p*-value		0.0303 *	0.011 *	0.9816	0.1767	0.2262
rs1107428594 (41134034)	AA	13	3.6333 ± 0.2937	5.4633 ± 0.2931	5.1862 ± 0.2885 ab	3.0320 ± 0.2985 a	6.7209 ± 0.2986
	AG	70	3.3233 ± 0.1256	5.3503 ± 0.1274	5.4742 ± 0.1241 a	2.2823 ± 0.1257 b	6.3155 ± 0.1256
	GG	70	3.3975 ± 0.1278	5.4442 ± 0.1288	5.1024 ± 0.1280 b	2.2546 ± 0.1336 b	6.5753 ± 0.1307
	*p*-value		0.6088	0.8577	0.1200	0.05 *	0.2334

The aggressive behavior indicators of each piglet with different genotypes of SNP rs1110730503 and rs1107428594 in porcine DRD2 gene were calculated using the GLIMMIX procedure in SAS. The data were presented as least square means (LS-Mean) ± standard errors (SE). Different letters (a, b) indicate that the difference is significant (*p* < 0.05). * *p* < 0.05

## Data Availability

The datasets generated and/or analyzed during the current study are available from the corresponding author upon reasonable request.

## Supplementary Materials

The following supporting information can be downloaded at: https://www.mdpi.com/article/10.3390/biology11010135/s1, Table S1: Total primers used and small RNA sequence, Table S2: Primer sequences for transcripts used in real-time quantitative PCR, Table S3: The promoter prediction of porcine dopamine receptor D2 (DRD2) gene by Promoter 2.0, Table S4: The promoter prediction of porcine DRD2 gene by Neural Network Promoter Prediction, Table S5: The CpG islands prediction of porcine DRD2 gene by Meth Primer. Complete Western blot images were obtained in supplementary materials. The uncropped western blot figures can be accessed in Supplementary files.
