# Supplementary material for: Functionally Antagonistic Transcription Factors IRF1 and IRF2 Regulate the Transcription of the Dopamine Receptor D2 Gene Associated with Aggressive Behavior of Weaned Pigs
Source: Biology (Basel). 2022 Jan 14;11(1):135. doi: 10.3390/biology11010135 (PMC8773180; doi:10.3390/biology11010135)
Supplement: Supplementary file 1 [file biology-11-00135-s001.zip › Supplementary Table.pdf]

Table S1 Total primers used and small RNA sequence

Name	Sequence (5'-3')
DRD2-P1-F	CGACGCGTCGGGGGTTGTGGAGGGATCGCT
DRD2-P2-F	CGACGCGTCGGGGCACCAGCTAGTAAGACG
DRD2-P3-F	CGACGCGTCGCTGGGAATGGGGGTGATGAT
DRD2-P-R	CCCAAGCTTGGGGCCAGGACCCAGCAAATCGG
PCR-DRD2-rs1110730503 -F	CGAGCGGGCAGAGTTTCCAA
PCR-DRD2-rs1110730503 -R	CCTCCCTCCTCGACTGAATC
PCR-DRD2-rs1107428594-F	GGAAGCTATCTGAGTGAGTC
PCR-DRD2-rs1107428594-R	CAGGATGAATCAGGAATAAG
DRD2-A/T-WT-F	CGACGCGTCGCAACTGCCCCATAGGGAGAA
DRD2-A/T-WT-R	CCCAAGCTTGGGGGAGAGTAGAGCGAGAACCA
DRD2-A/T-MUT-F	CTCCACATTTAaGTGACTTGAGACTTTTCAGCTTCTAAG
DRD2-A/T-MUT-R	GTCACtTAAATGTGGAGATGCGAAAACAACCC
IRF1-WT-F	CGACGCGTCGCCATCTTAGGTCAATTCCAA
IRF1-WT-R	CCCAAGCTTGGGACCCCAAAGTAACACCCTAT
IRF1-MUT-F	TCgggaaCTGACCTCTTGGATGCAAAGTGC
IRF1-MUT-R	AAGAGGTCAGttcccGAGGAGCCTGGTCCAAGGAC
IRF2-WT-F	CGACGCGTCGGGGCACCAGCTAGTAAGACG
IRF2-WT-R	CCCAAGCTTGGGGCCAGGACCCAGCAAATCGG
IRF2-MUT-F	CTCCACATTTcctgtACTTGAGACTTTTCAGCTTCTAAGGA
IRF2-MUT-R	GTacaggAAATGTGGAGATGCGAAAACAA
ChIP-IRF1-F	AGCACAGAACTAACCAAAGG
ChIP-IRF1-R	TTTGAGGAGTCAGAGTGTAG
ChIP-IRF2-F	CGAGCGGGCAGAGTTTCCAA
ChIP-IRF2-R	CGGCACATTAGGGTGTAGGA

ChIP-Non-specific-F	GGATTCAGTCTGGAAGGAGC
ChIP-Non-specific-R	GCAGGTCAGAAAATACGAGC
siIRF1-1 sense	GGGCUGAUCUGGAUUAAUATT
siIRF1-1 antisense	UAUUAAUCCAGAUCAGCCCTT
siIRF1-2 sense	CUCCCUGCCAGAUAUUGAATT
siIRF1-2 antisense	UUCAAUAUCUGGCAGGGAGTT
siIRF2-1 sense	GGGCUUAGUAAUGGAGUAATT
siIRF2-1 antisense	UUACUCCAUUACUAAGCCCTT
siIRF2-2 sense	GCACCACUCUUUAGAAACUTT
siIRF2-2 antisense	AGUUUCUAAAGAGUGGUGCTT

Table S2 Primer sequences for transcripts used in real-time quantitative PCR

Gene		Primer sequence (5′→3′)	Tm	Product length (bp)	amplification efficiency
BAX	Forward:	CCGAAATGTTTGCTGACG	87	154	102%
	Reverse:	AGCCGATCTCGAAGGAAGT			
BCL-2	Forward:	TTCTTTGAGTTCGGTGGGG	87	195	101%
	Reverse:	CCAGGAGAAATCAAATAGAGGC			
DRD2	Forward:	TGGCAAAACCCGGACCTCCC	87	227	105%
	Reverse:	TGGGGTTCACGGCGCTGTTG			
IRF1	Forward:	GTGAAGGACCAGAGCAGGAA	87	276	107%
	Reverse:	TGACAGTGCTGGAGTAAGGG			
IRF2	Forward:	TGGACAGCACCATTGAGGAT	88	304	108%
	Reverse:	TTGGAAGTGACGAAGGTTGC			
GAPDH	Forward:	GATGGTGAAGGTCGGAGTG	88	446	107%
	Reverse:	CGAAGTTGTCATGGATGACC			

Table S3 The promoter prediction of porcine dopamine receptor D2 (*DRD2*) gene by Promoter 2.0

	position	score	likelihood
DRD2	400（-1900）	0.545	Marginal prediction
	1800(-500)	0.577	Marginal prediction

Table S4 The promoter prediction of porcine *DRD2* gene by Neural Network Promoter Prediction

	Start	End	Score	Promoter Sequence
DRD2	432(-1818)	482-1768)	0.91	GGGGCGGGGCCTGTCTCAGGGGCGGGGCCCGAGGCGCCCCATGGCCCCGC
	448(-182)	498(-1752)	0.88	CAGGGGCGGGGCCCGAGGCGCCCCATGGCCCCGCCCCCTCCCTCCCGCGC
	573(-1677)	623(-1627)	0.84	CTCCGCGCCGGGTCCCTGCGCCTCACGCCCGCCCCGCGCCGGCCCCGCCC
	584(-1666)	634(-1616)	0.81	GTCCCTGCGCCTCACGCCCGCCCCGCGCCGGCCCCGCCCCACCCCGCCCC
	607(-1643)	657(-1593)	0.87	CGCGCCGGCCCCGCCCCACCCCGCCCCGCCGCGGCCGGTCCACTGCTCCC
	636(-1614)	686(-1564)	0.82	CGCGGCCGGTCCACTGCTCCCAGCGGGTCCGAGCCGGCCGAGCGGCTGCC
	683-1567)	733(-1517)	0.89	GCCCGCCGGGGCTCTGAACGGCGCGGCGGGGCCGGGAGCCAGGGACCCGC

Table S5 The CpG islands prediction of porcine *DRD2* gene by Meth Primer

	Size(bp)	Start	End
**Island 1**	507	356（-1894）	862（-1388）
**Island 2**	421	874（-1376）	1294（-956）
**Island 3**	106	1391（-859）	1496（-754）
**Island 4**	115	1597（-653）	1711（-539）
