# Supplementary figures and images for: Functionally Antagonistic Transcription Factors IRF1 and IRF2 Regulate the Transcription of the Dopamine Receptor D2 Gene Associated with Aggressive Behavior of Weaned Pigs
Source: Biology (Basel). 2022 Jan 14;11(1):135. doi: 10.3390/biology11010135 (PMC8773180; doi:10.3390/biology11010135)

Figure S1

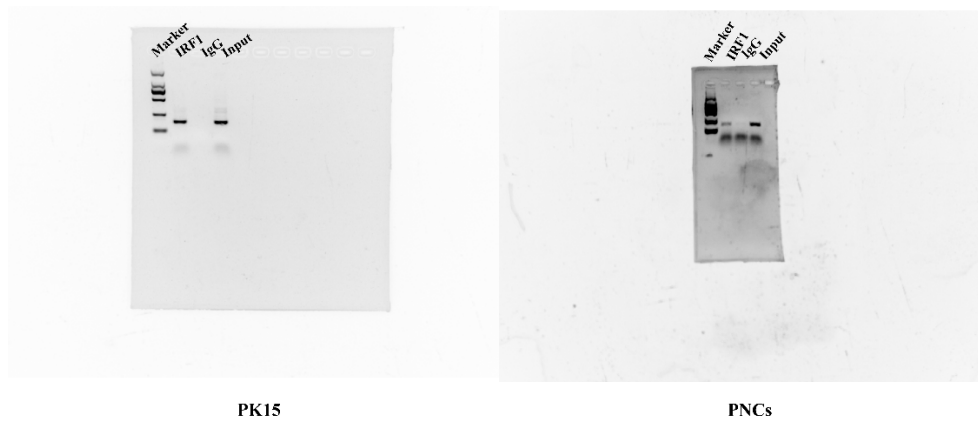

Figure S2

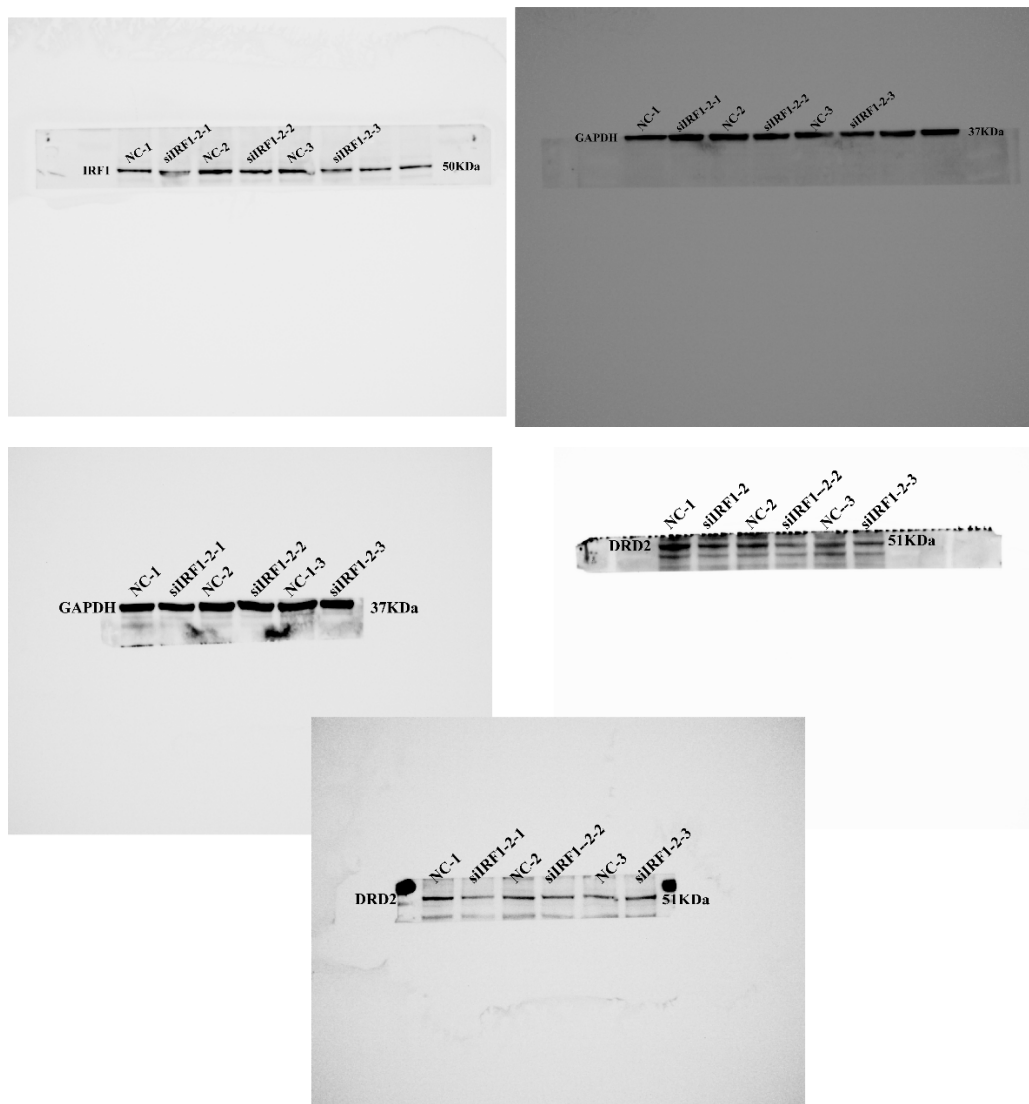

Figure S3

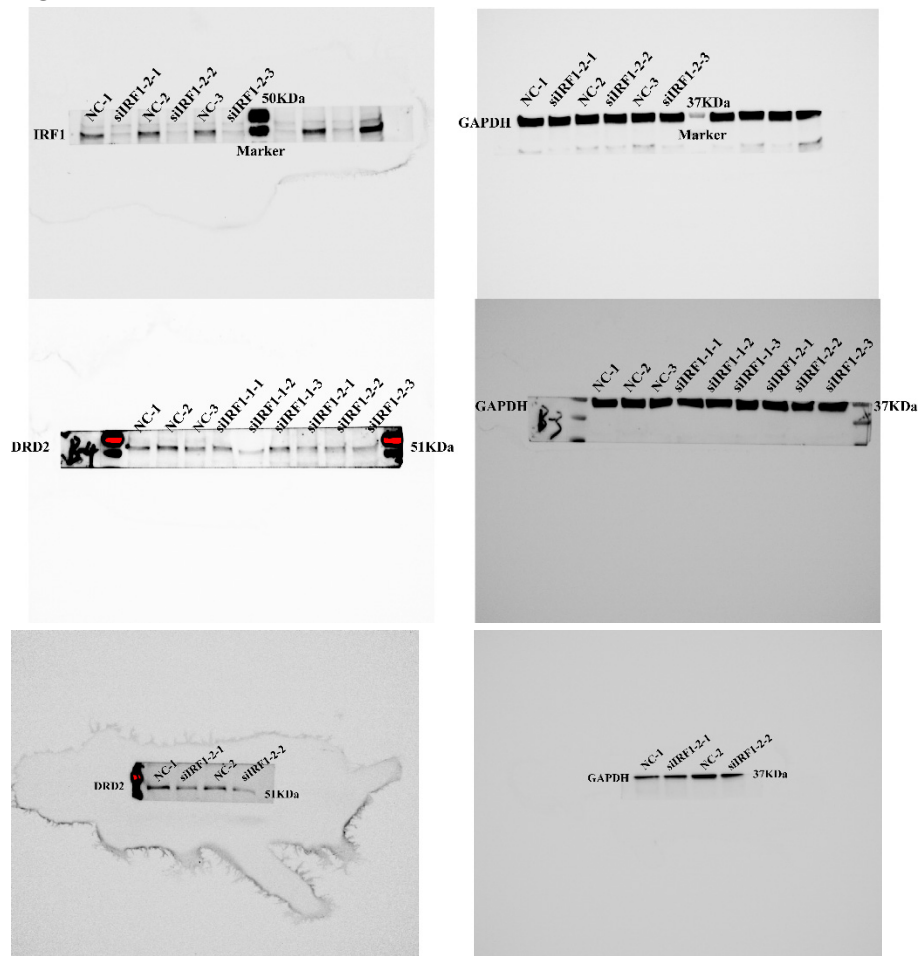

Figure S4

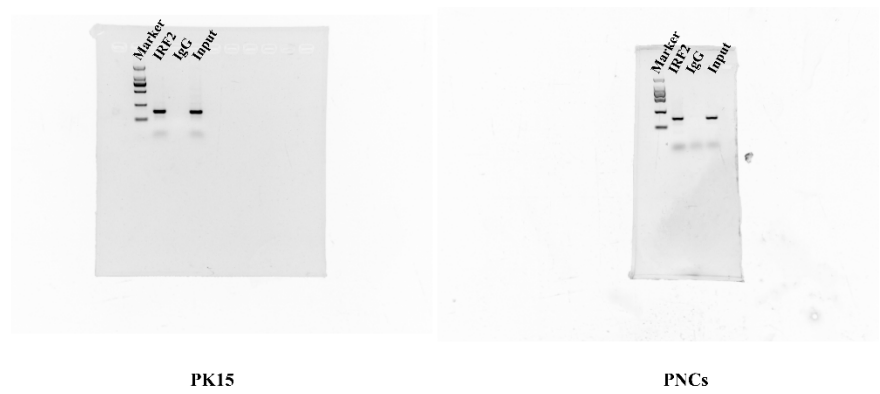

Figure S5

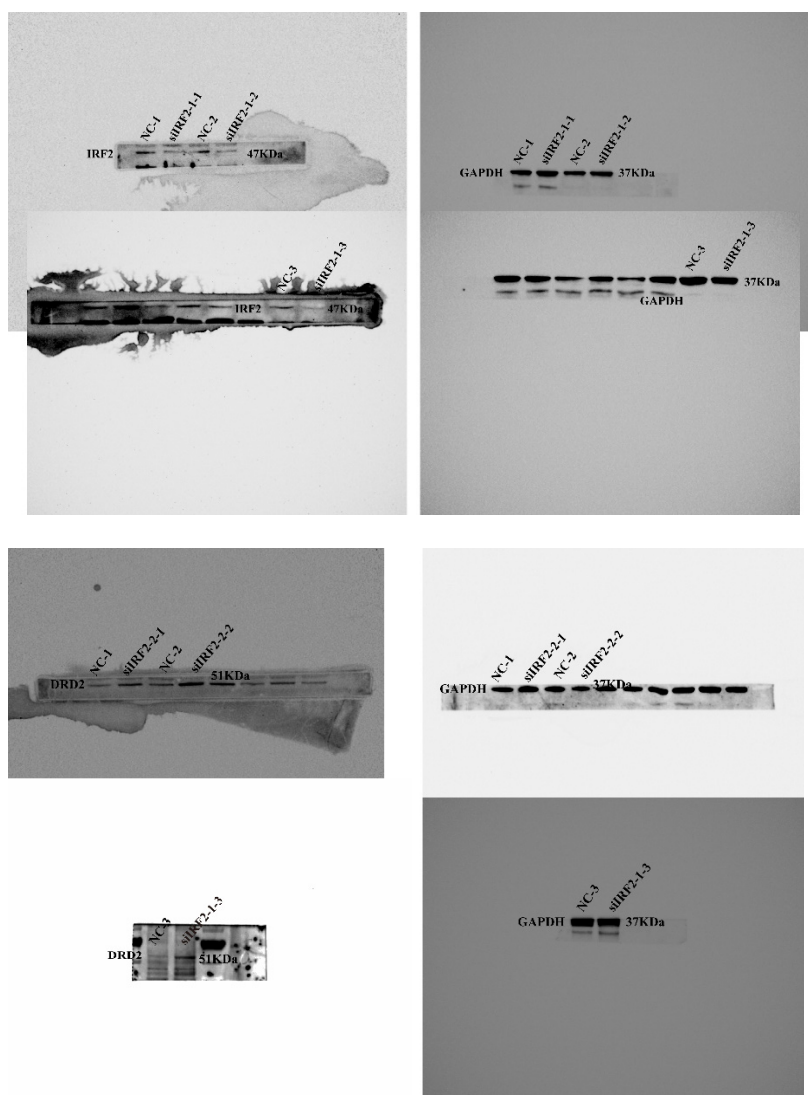

Figure S6

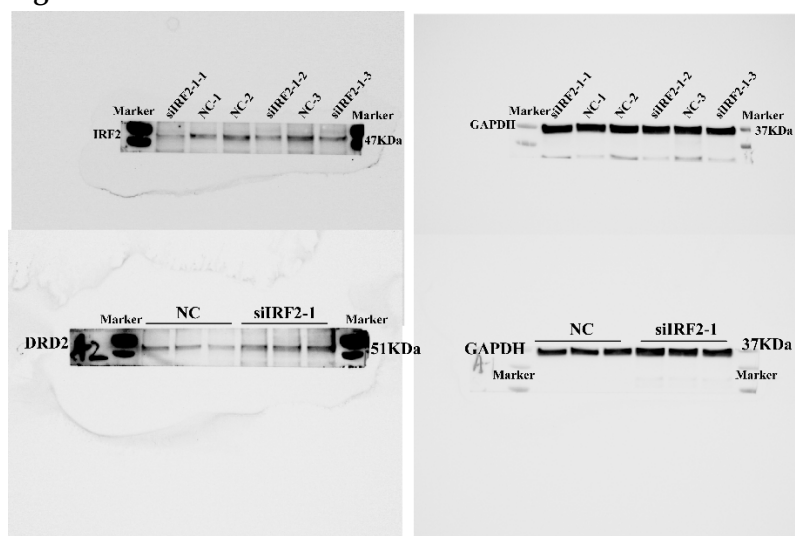

Figure S7

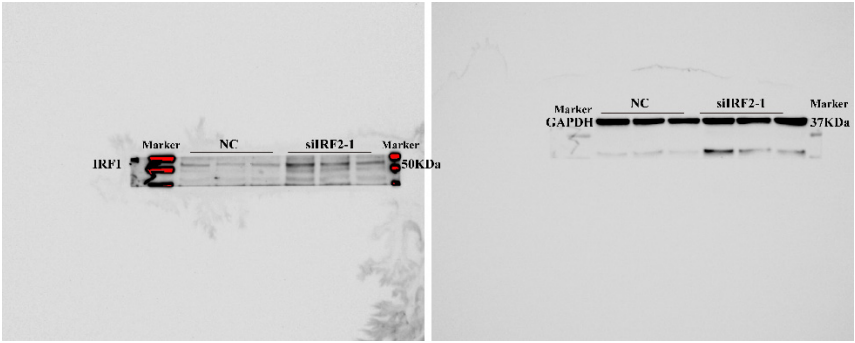

Figure S8

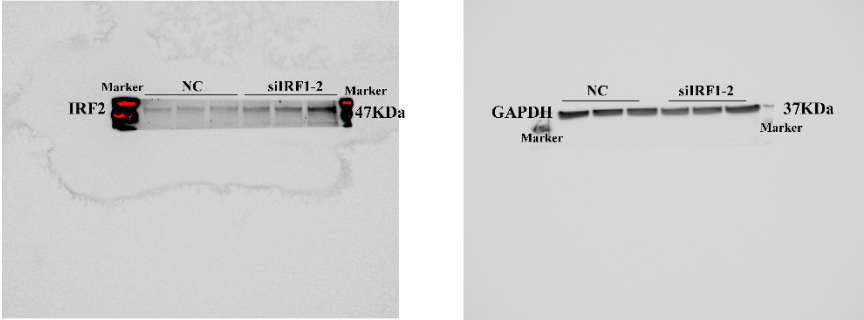

Supplement: Supplementary file 1 [file biology-11-00135-s001.zip › Supplementary figure.pdf]
